# Methylomic biomarkers of lithium response in bipolar disorder: a clinical utility study

**DOI:** 10.1186/s40345-023-00296-6

**Published:** 2023-04-29

**Authors:** C. Marie-Claire, C. Courtin, F. Bellivier, S. Gard, M. Leboyer, J. Scott, B. Etain

**Affiliations:** 1 Inserm UMR-S 1144, Optimisation Thérapeutique en Neuropsychopharmacologie, Université Paris Cité, 4 Avenue de l’observatoire, 75006 Paris, France; 2 Département de Psychiatrie et de Médecine AddictologiqueHôpitaux Lariboisière-Fernand Widal, GHU APHP.Nord Université de Paris, 75010 Paris, France; 3 Fondation Fondamental, 94010 Créteil, France; 4 Centre Hospitalier Charles Perrens, Pôle de Psychiatrie Générale et Universitaire, Bordeaux, France; 5 Translational Neuro-Psychiatry, Université Paris Est Créteil, INSERM U955, IMRB, Créteil, France; 6 Département Médico-Universitaire de Psychiatrie et d‘Addictologie (DMU IMPACT), Fédération Hospitalo-Universitaire de Médecine de Précision en Psychiatrie (FHU ADAPT), AP-HP, Hôpitaux Universitaires Henri Mondor, Créteil, France; 7 Institute of Neuroscience, Newcastle University, Newcastle Upon Tyne, NE4 5PL UK

**Keywords:** DNA methylation, Bipolar disorders, Lithium, MS-HRM, Biomarker, Transferability

## Abstract

**Background:**

Response to lithium (Li) is highly variable in bipolar disorders (BD). Despite decades of research, no clinical predictor(s) of response to Li prophylaxis have been consistently identified. Recently, we developed epigenetic Methylation Specific High-Resolution Melting (MS-HRM) assays able to discriminate good responders (GR) from non-responders (NR) to Li in individuals with BD type 1 (BD-I). This study examined whether a combination of clinical and epigenetic markers can distinguish NR from other types of Li responders.

**Methods:**

We recorded clinical variables that are potentially associated with Li response in 64 individuals with BD-I. MS-HRM assays were performed on DNA isolated from peripheral blood. We used backward stepwise logistic regression analyses, followed by receiver operating characteristic (ROC) curve analysis to estimate the performance of the clinical variables, alone then in combination with the epigenetic biomarkers, to identify GR and partial responders (PaR) vs NR.

**Results:**

Polarity at onset, psychotic symptoms at onset and family history of BD classified correctly 70% of individuals according to their Li response (PaR + GR = 86%; NR = 35%). When combined with the epigenetic biomarkers, these three clinical variables plus alcohol misuse (and one DMR: Differentially Methylated Region) correctly classified 86% of individuals, improving the prediction of PaR + GR (93%) and of NR (70%). The ROC analysis demonstrated an improvement in the area under the curve from 0.75 (clinical variables alone) to 0.87 (combination of clinical and epigenetic markers).

**Conclusions:**

Combining clinical predictors and DNA methylation markers of Li response may have greater utility in clinical practice than relying on clinical characteristics alone.

**Supplementary Information:**

The online version contains supplementary material available at 10.1186/s40345-023-00296-6.

## Introduction

Bipolar disorder (BD) is a highly prevalent psychiatric disorder that is characterized by the recurrence of major depressive and (hypo)manic episodes interspersed by periods of normothymia. BD is one of the most burdensome disorders worldwide in terms of disability-adjusted life-year in both working age adults and 12–24 year olds (Collins et al. 2011; Gore et al. 2011). Lithium (Li) is the first-line prophylactic treatment for BD and has proven efficacy not only for treating acute manic episodes, but also for preventing mood episode relapses, recurrences of all polarities (Can et al. 2014; Geddes et al. 2004; Miura et al. 2014). In addition, a recent review of the literature on suicidal behavior prevention in BD concluded that lithium treatment as a suicide-preventing therapeutics is the best option (Tondo et al. 2021). However, response to Li is heterogeneous among individuals with BD and three subpopulations (good responders (GR), partial responders (PaR) and non-responders (NR)) have been repeatedly identified, with around one third of the individuals belonging to each group (Kessing et al. 2011; Manchia et al. 2013).

A systematic review and meta-analysis (Hui et al. 2019) identified six predictors of GR: mania-depression-interval sequence, absence of rapid cycling, absence of psychotic symptoms, family history of BD, shorter pre-Li illness duration and later age of onset. A further systematic review sociodemographic and/or clinical variables associated with Li response identified two factors were associated with GR: good social support and episodic evolution of BD, while four factors were associated with poor response: alcohol use disorder; personality disorders; higher lifetime number of hospital admissions and rapid cycling pattern (Grillault-Laroche et al. 2020). However, when the methodological quality and heterogeneity of included studies was accounted for, only one factor was robustly associated with Li non-response: higher lifetime number of hospitalization admissions (Grillault-Laroche et al. 2020). As such, it appears that clinical characteristics are insufficient for reliable prediction of Li response in day to day practice. In this context, the identification of biological markers that may substantially increase the predictability of Li response (when combined with socio-demographic and clinical variables) represents an important step towards the application of personalized medicine in psychiatry.

Family studies have suggested a heritability of Li response (Papiol et al. 2022) and the search for genetic markers is a very active field. The International Consortium on Lithium Genetic (ConLiGen) has published the largest genome wide association study available to date, and identified two long non coding RNAs as associated with Li response in BD (Hou et al. 2016). Another study identified a total of 137 genes associated with Li response (Nunes et al. 2021). Beyond genetic markers, epigenetic biomarkers have received more recent attention for the study of treatment outcomes in BD (Legrand et al. 2021; Marie-Claire et al. 2021). Using a genome-wide analysis of DNA methylation profiles among individuals with BD type 1 (BD-I), we recently identified an epigenetic signature that combines seven differentially methylated regions (DMRs) and accurately discriminates GR from NR to Li (Marie-Claire et al. 2020). To validate these findings and allow potential transfer to the bedside, we then employed an easy to implement, cost and time efficient method: Methylation Specific High-Resolution Melting (MS-HRM) (Šestáková et al. 2019). MS-HRM assays have been developed, with three of the previously identified DMRs to validate the performance of these markers. We have replicated the preliminary findings in an extended sample showing that MS-HRM-measured DMRs correctly classified up to 84% of individuals as GR or NR (Marie-Claire et al. 2022).

The present study represents an exploratory analysis of the clinical utility of methylomic MS-HRM assays in combination with clinical factors to discriminate individuals who have benefited from Li (GR and PaR) from those who did not (NR).

## Materials and methods

This study was approved by the Research Ethics Board of the Pitié-Salpétrière Hospital, Paris—France and is registered under the Clinical trial number NTC02627404.

### Participants

The sample comprised 64 individuals who met the study eligibility criteria. The main inclusion criteria were a diagnosis of BD type I according to DSM-IV criteria (4th edition of the Diagnostic and Statistical Manual of Mental Disorders), currently in remission (i.e. no major episode or hospitalization in the last six months) and euthymic (i.e., a score of < 8 on both the Montgomery-Åsberg Depression Rating Scale (Montgomery and Åsberg 1979) and the Mania Rating Scale (Bech et al. 1978) and being willing and able to give written informed consent. Individuals with a current diagnosis of comorbid alcohol or cannabis abuse or dependency were excluded.

Eligible participants were interviewed with the French version of the Diagnostic Interview for Genetic Studies (DIGS) to confirm the diagnosis of BD and to assess lifetime comorbidities (such as alcohol and cannabis use) and with the Family Interview for Genetic Studies (FIGS) to assess family history (Nurnberger et al. 1994). All participants were currently on a stable prophylaxis medication (i.e., no change in the last six months) and are completely independent from those previously published (Marie-Claire et al. 2022, 2020).

### Phenotyping of response to lithium

Response to Li was rated using the “Retrospective Criteria of Long-Term Treatment Response in Research Subjects with Bipolar Disorder”, also referred to as the “Alda scale” (Grof et al. 2002). This scale was specifically developed to allow a retrospective assessment of prophylactic response to treatment in naturalistic conditions (and considers both overall response to the introduction of Li on a 0–10 scale, whilst accounting for confounders of response such as poor adherence, etc.). In accordance with the available literature (Manchia et al. 2013), patients with total score >  = 7 were characterized as good responders (GR) and patients with total score ≤ 3 were characterized as non-responders (NR). Remaining individuals were classified as partial responders (PaR).

### DNA isolation and bisulfite modification

DNA was isolated from total peripheral blood collected at inclusion. Genomic DNA was extracted using the automated Maxwell 16 DNA Purification Instrument and the dedicated RSC Blood DNA Kit (Promega). All DNA samples were stored at − 80 °C at the “Plateforme de Ressources Biologiques des Hôpitaux Universitaires Henri Mondor in Creteil France”. As previously described, genomic DNA input was 200 ng to be modified with sodium bisulfite using the EZ DNA^™^ methylation kit (Zymo Research, Irvine, CA, USA). Human methylated and unmethylated DNA standards were diluted before bisulfite conversion to prepare 0, 10, 20, 30, 40, 50, 60, 70, 80, 90 and 100% methylated to unmethylated template ratios. Bisulfite modified DNA was eluted in 10 µL of nuclease-free water according to the manufacturer’s instructions. The modified DNA was quantified with a NanoDrop One Spectrophotometer (Ozyme, Saint-Cyr-l’École, France).

### Methylation sensitive high-resolution melting

Methylation-Sensitive High-Resolution Melting (MS-HRM) is based on the comparison of the melting profiles of PCR products from unknown samples with profiles specific for PCR products derived from methylated and unmethylated control DNAs (Šestáková et al. 2019). As previously described, MS-HRM tests have been designed to amplify three previously identified DMRs in GR as compared to NR individuals with BD-I. Primers design with the Bisearch online tool (http://bisearch.enzim.hu/) and amplification details for DMR17107 (amplicon size 169 bp), DMR106540 (amplicon size 116 bp) and DMR24332 (amplicon size 147 bp) have been previously described (Marie-Claire et al. 2022). PCR reactions were performed on a CFX384 Touch Real-Time PCR Detection System (Biorad Laboratories, Des Plaines, IL, USA) in a final volume of 10 µL, containing 200 nM of each primer, 5 µL of Precision Melt Supermix (Biorad Laboratories) and 10 ng of bisulfite treated DNA. The initial denaturation (95°, 3 min) was followed by 45 cycles of 10 s at 95 °C, 30 s at 50 °C, 30 s at 72 °C. The HRM step consisted of a denaturation of all products at 95 °C for 30 s followed by an annealing at 60 °C for 1 min. Samples were then slowly warmed to 95 °C at 0.2 °C per second, holding for 10 s after each stepwise increment and fluorescence data were collected. Each sample was analyzed in triplicate. For the DNA methylation assessment, bisulfite converted dilutions of methylated and unmethylated DNA standards were analyzed together with the samples. Peak-heights were calculated automatically with the CFX Maestro Software (Version 2.2; Bio-Rad Laboratories, Inc., Hercules, CA, USA). Linear curves of the peak-heights of the Tm first derivative of HRM curves against the methylation percentage of the standard were plotted (Tse et al. 2011). The lab technician was blinded to response status of the participants.

### Statistical analysis

All statistical analyses were performed with IBM SPSS version 28 software. A p-value < 0.05 was considered statistically significant.

We performed backward stepwise logistic regression analyses (BSLR), followed by receiver operating characteristic (ROC) curve analysis with findings reported as the estimated area under the curve (AUC) with 95% confidence intervals (95% CI). The following available variables were included in model 1 of the BSLR (socio-demographic and clinical variables alone): age, sex, cigarette smoking status, lifetime number of hospitalizations, age at onset of BD, polarity at onset, psychotic symptoms at onset, family history of BD, lifetime alcohol misuse, lifetime cannabis misuse, panic disorders, Li prescribed as the first mood stabilizer (vs 2nd or 3rd choice, etc.). The BSLR analysis was then repeated, but the model included sociodemographic and clinical variables alongside the three DMRs (model 2). The following variables were not used into the models since redundant with some B items of the Alda scale: number of episodes (B1 item) and frequency of episodes, including rapid cycling (B2 item). Since there are different ways to use the Alda scale as a complement of the classical three groups (i.e. using A-scale, or A-scale with B score < 4 for instance), we also provide an analysis based on model 2, but using the A-scale only in a linear regression. Multicollinearity was examined using the Variance Inflation Factors (VIF) (a VIF above 4 indicates that multicollinearity might exist).

## Results

### Sample characteristics

As shown in Table 1, the 64 cases with BD-I comprised 44 PaR + GR and 20 NR. The groups did not differ significantly in current age (p = 0.91) or sex distribution (p = 0.97). Groups showed similar clinical characteristics except that, compared with the NR group, there were non-significant trends for the PaR + GR group to have more first-degree relatives with BD (p = 0.09), more frequent manic onset of BD (p = 0.06), and less frequent lifetime cannabis misuse (p = 0.09).

**Table 1:** Socio‑demographic and clinical characteristics of the sample (N = 64)

	PaR + GR n = 44	NR N = 20	p*
	N (%) or median (IQR)	N (%) or median (IQR)	
Socio-demographics			
Age at inclusion	42 (32–50)	43 (32–49)	0.91
Sex (females)	24 (54%)	11 (55%)	0.97
Family history			
First-degrees relatives with BD	21 (48%)	5 (25%)	0.09
Mode of onset			
Age at BD onset	23 (18–31)	25 (18–32)	0.83
Psychotic symptoms at onset	11 (25%)	7 (35%)	0.41
Manic polarity at onset	22 (50%)	5 (25%)	0.06
BD Course			
Rapid cycling	6 (14%)	1 (5%)	0.42
Duration of the illness	16 (9–24)	14 (8–20)	0.45
Lifetime number of episodes	8 (5–10)	8.5 (5–11)	0.49
Lifetime number of hospitalizations	4 (2–6)	4 (3–7)	0.26
Lithium as first prescribed stabilizer	27 (61%)	10 (50%)	0.42
Comorbidities			
Current tobacco use	26 (59%)	12 (60%)	0.94
Lifetime alcohol misuse	9 (20%)	4 (20%)	0.62
Lifetime cannabis misuse	4 (9%)	5 (25%)	0.09
Lifetime panic disorders	6 (14%)	4 (20%)	0.54

^*^Chi-square or Exact Fisher or Mann–Whitney tests when appropriate

### Association between Li response and, clinical variables alone or in combination with DMRs

**Fig. 1:**
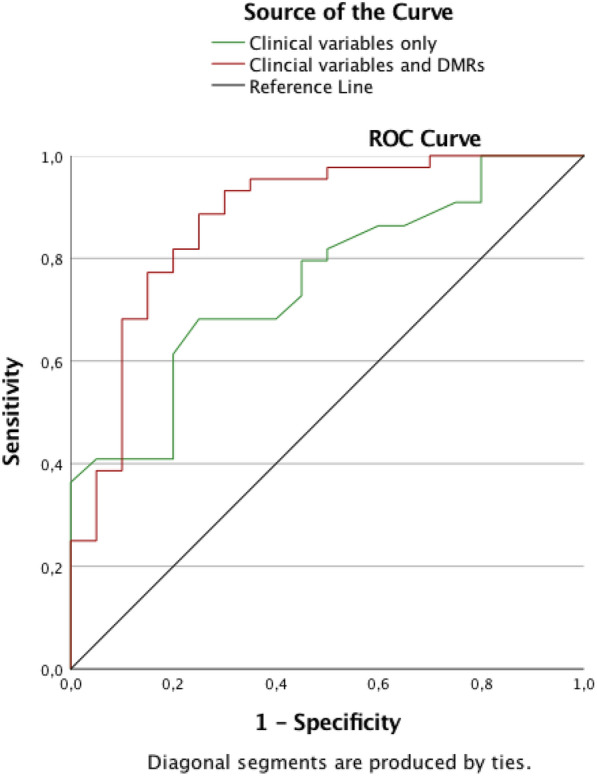
Prediction of Li response for clinical variables alone or combined with DMRs percentage of methylation

Model 1: The BSLR model including only the clinical variables identified a set of three potential predictors of response to Li: family history of BD (p = 0.03), polarity at onset (p = 0.02) and psychotic symptoms at onset (p = 0.04) (Table 2). The Nagelkerke R^2^ was 0.27. This model correctly classified 70% of individuals (86% of Par + GR, but only 35% of NR). As shown in Fig. 1, the AUC was 0.75 (95% CI 0.63–0.87).

**Table 2:** Model 1: Association between good and partial response to lithium, socio-demographic and clinical variables

Variables	Beta	SE	Wald	df	p	VIF
Polarity at onset	− 1.876	0.807	5.402	1	0.020	1.555
Family history of BD	1.511	0.704	4.602	1	0.032	1.161
Psychotic symptoms at onset	− 1.728	0.831	4.330	1	0.037	1.642
Lifetime number of hospitalizations	− 0.108	0.072	2.242	1	0.134	1.078
Constant	− 0.422	0.912	0.214	1	0.644	

Variables ordered from lowest to highest p values

VIF: Variance inflation factor

Model 2: This model identified a set of four associated clinical variables: family history of BD (p = 0.04), polarity at onset (p = 0.04), psychotic symptoms at onset (p = 0.01) and lifetime alcohol misuse (p = 0.03), in addition to DMR24332 (p = 0.007) (Table 3). The Nagelkerke R^2^ was 0.50. As shown in the ROC curves in Fig. 1, model 2 correctly classified 86% of individuals (93% of Par + GR and 70% of NR) with an AUC of 0.87 (95% CI 0.77–0.97).

**Table 3:** Model 2: association between good and partial response to lithium, socio-demographic and clinical variables and DMRs

Variables	Beta	SE	Wald	df	p	VIF
DMR24332	− 0.248	0.093	7.167	1	**0.007**	3.461
Psychotic symptoms at onset	− 3.056	1.228	6.189	1	**0.013**	2.744
Lifetime alcohol misuse	3.132	1.489	4.426	1	**0.035**	2.956
Family history of BD	1.918	0.915	4.395	1	**0.036**	1.554
Polarity at onset	− 2.311	1.125	4.217	1	**0.040**	2.446
Lifetime number of hospitalizations	− 0.197	0.109	3.287	1	0.070	1.951
Smoking status	− 1.998	1.114	3.219	1	0.073	2.534
DMR106540	− 0.268	0.152	3.089	1	0.079	1.987
Lithium as first MS	− 0.730	0.448	2.662	1	0.103	1.689
Lifetime cannabis misuse	1.555	1.061	2.146	1	0.143	1.505
Constant	30.983	14.192	4.766	1	0.029	

Variables ordered from lowest to highest p values

VIF: Variance inflation factor

Using the same variables as in model 2 but using the A scale as the dependent variable confirmed the association between response to lithium and DMR24332 (p = 0.011) and Lifetime number of hospitalizations (p = 0.002). Other variables identified in model 2 were no longer associated with response to lithium. Results are described in Additional file 1: Table S1.

Finally, PaR + GR and NR were—by definition—different on the item B5 (p = 0.005), because in particular NR were more likely to be treated with lithium plus other mood stabilizers. Therefore, we ran model 2 again but including the item B5 as a potential confounding factor. In this model, DMR24332 (p = 0.0011) was still associated with response to lithium (Additional file 2: Table S2).

## Discussion

Several clinical factors have been associated with Li response in BD. However, despite decades of research, there is still no identified predictors of lithium treatment response usable in clinical practice. The goal of this preliminary study was to examine whether the combination of clinical and epigenetic markers might be of clinical utility to better discriminate individuals classified as GR/PaR from NR to Li treatment. We showed that, in a sample of individuals with BD-I, a combination of four clinical variables (family history of BD, polarity and psychotic symptoms at onset and lifetime alcohol misuse) and one epigenetic biomarker identified correctly 93% of PaR + GR and 70% of NR. This is the first study to provide evidence that combining clinical variables and epigenetic biomarkers may accurately distinguish individuals with BD-I who will show a poor response to Li (i.e., NR) from who will show at least some clinically meaningful benefit (i.e., PaR or GR).

Although Li is the first-line acute and maintenance treatment for BD, the observed variability in benefits (response) and negative experiences (side-effects) represents a challenge for clinicians who aim to better tailor treatment to each individual, ensuring timely prescription of the ‘right drug at the right time for the right patient’. Our findings indicate that, when routinely recorded clinical characteristics are examined alone, three variables (BD polarity at onset, family history of BD and psychotic symptoms at onset) were significantly associated with response to Li in our sample. In the last published meta-analysis, family history of BD was found associated with Li response (Odds Ratio (OR): 1.61, p = 0.036), while no significant evidence with manic index episode was found (Hui et al. 2019). The association between Li response and psychotic symptoms is less clear cut, with some studies suggesting that it is a lifetime pattern of psychotic symptoms rather than the presence of psychotic symptoms at first onset of BD that may be more useful as a predictor (Hui et al. 2019; Scott et al. 2020a).

In the current study, we suggest that combining four commonly recorded clinical variables (family history, psychotic onset and onset polarity plus lifetime history of alcohol misuse) with DNA methylation biomarkers slightly improve the classification of those who will benefit from lithium treatment (GR + PaR) from 86 to 93%. While the contribution of epigenetic biomarkers increased markedly the classification of NR (from 35 to 70%), therefore allowing to better target patients for whom the benefit/risk ratio is not favorable to lithium treatment. This is in line with our previous results showing that these MS-HRM assays performed very well in identifying NRs (Marie-Claire et al. 2022). If confirmed in further studies, this could avoid subjecting these individuals to a trial of 18–24 months of Li during which they are exposed to the risk of side effects.

Among the three tested DMRs, only one remained significant in combination with the three clinical markers. This DMR24332 is located in an intron of *LINC01237*, a Long Intergenic Non-Protein Coding RNA which makes it difficult to draw any conclusion regarding the biological significance. Nonetheless, its inclusion in a combination strategy with clinical factors is suggested to be relevant to characterize response to Li in DNA from whole blood from individuals with BD-I.

We acknowledge several limitations. Although this sample is independent of those reported in our previous publication (Marie-Claire et al. 2022), the total number of participants, and relatively small size of the NR subgroup, may have limited our ability to identify other relevant clinical or epigenetic predictors of Li response. We did not apply any correction for multiple testing in this exploratory study, the present results should therefore be extended in larger independent samples of individuals with BD-I. Third, the choice of studied clinical variables might also be questioned. However, they were selected on the basis of the last meta-analysis (Hui et al. 2019), our previous review on the socio-demographic and clinical predictors of Li response in BD (Grillault-Laroche et al. 2020) and their availability in the database. Third, the study population is cross-sectional which does not allow to determine whether the observed methylation differences were induced by Li, other medications and/or by disease course that might differ between GRs, PaRs and NRs. Moreover, in the available database, we do not have any information regarding currently prescribed mood stabilizers other than lithium. For most individuals (N = 49), lithium was prescribed as the sole mood stabilizers or only with low dose of antidepressants or antipsychotics (based on the values of the B5 item) but we cannot exclude that other medications might contribute to the observed results. However, introducing the B5 item in the final model did not modify the significant association between DMR24332 and response status suggesting that the level of comedications was unlikely to skew the results (Additional file 2: Table S2). Fourth, the findings apply only to individuals with BD-I since we did not included individuals with the more heterogeneous clinical presentations of BD such as BD type 2 or bipolar spectrum disorders. Furthermore, our analyses were based on the Alda scale’s total score. However, our group have examined the quality and performance of this scale and proposed several improvements (Scott et al. 2021, 2020b, 2017). Therefore, the present results should be replicated using different ways to use the Alda scale. Finally, the ROC data are presented as indicative because prediction models should be evaluated in unseen data and cross-validation would be required to confirm our results. These weaknesses highlight that replication and extension of the findings is required in larger independent samples of individuals with BD-I and with a characterization of their response to lithium using the Alda Scale.

In conclusion, the present findings reinforce the potential transferability of epigenetic results obtained in research laboratories towards clinical practice settings and validate a combined strategy with clinical and DNA methylation markers. This is a promising step to improve the identification of individuals that will or will not benefit from this treatment in order to optimize their medical care. Future studies should be performed on larger, independent and clinically well-characterized samples to use more complex statistical approaches and/or different ways to use the Alda scale.

## Supplementary Information

**Additional file 1: Table S1.** Association between response to lithium Using Alda A score, socio-demographic and clinical variables and DMRs.

**Additional file 2: Table S2.** Association between good and partial response to lithium, socio-demographic and clinical variables (including the values of the B5 item) and DMRs.

## Data Availability

Due to ethical and legal restrictions, data involving clinical participants cannot be made publicly available. All relevant data are available upon request to the authors for researchers who meet the criteria for access to confidential data.
