# Supplementary material for: Methylomic biomarkers of lithium response in bipolar disorder: a clinical utility study
Source: Int J Bipolar Disord. 2023 Apr 29;11:16. doi: 10.1186/s40345-023-00296-6 (PMC10148930; doi:10.1186/s40345-023-00296-6)
Supplement: Supplementary file 1 — Additional file 1: Table S1. Association between response to lithium Using Alda A score, socio-demographic and clinical variables and DMRs. [file 40345_2023_296_MOESM1_ESM.docx]

**Supplementary Table S1**: Model 2: Association between response to lithium Using Alda A score, socio-demographic and clinical variables and DMRs

|  | **Beta** | **SE** | **t** | **p** | **VIF** |
| --- | --- | --- | --- | --- | --- |
|  |  |  |  |  |  |
| DMR24332 | -0.144 | 0.055 | -2.631 | **0.011** | 1.704 |
| Psychotic symptoms at onset | -0.532 | 0.777 | -0.685 | 0.496 | 1.231 |
| Lifetime alcohol misuse | 1.104 | 0.967 | 1.142 | 0.259 | 1.523 |
| Family history of BD | 1.125 | 0.682 | 1.651 | 0.105 | 1.129 |
| Polarity at onset | -0.922 | 0.821 | -1.124 | 0.266 | 1.651 |
| Lifetime number of hospitalizations | -0.282 | 0.086 | -3.279 | **0.002** | 1.169 |
| Smoking status | -1.221 | 0.728 | -1.676 | 0.101 | 1.287 |
| DMR106540 | -0.136 | 0.094 | -1.447 | 0.154 | 1.438 |
| Lithium as first MS | 0.311 | 0.363 | 0.854 | 0.397 | 1.291 |
| Lifetime cannabis misuse | -0.764 | 1.017 | -0.752 | 0.456 | 1.258 |
| Constant | 26.891 | 7.768 | 3.462 | 0.001 |  |

Variables ordered from lowest to highest p values

VIF: Variance Inflation Factor
