# Supplementary material for: Methylomic biomarkers of lithium response in bipolar disorder: a clinical utility study
Source: Int J Bipolar Disord. 2023 Apr 29;11:16. doi: 10.1186/s40345-023-00296-6 (PMC10148930; doi:10.1186/s40345-023-00296-6)
Supplement: Supplementary file 2 — Additional file 2: Table S2. Association between good and partial response to lithium, socio-demographic and clinical variables (including the values of the B5 item) and DMRs. [file 40345_2023_296_MOESM2_ESM.docx]

**Supplementary Table S2** : Association between good and partial response to lithium, socio-demographic and clinical variables (including the values of the B5 item) and DMRs

| **Variables** | **Beta** | **SE** | **Wald** | **df** | **p** |
| --- | --- | --- | --- | --- | --- |
| DMR24332 | -0.260 | 0.102 | 6.507 | 1 | **0.011** |
| Psychotic symptoms at onset | 3.429 | 1.374 | 6.226 | 1 | **0.013** |
| B5 item | -1.420 | 0.580 | 5.992 | 1 | **0.014** |
| Family history of BD | -2.619 | 1.170 | 5.010 | 1 | **0.025** |
| Smoking status | 2.866 | 1.407 | 4.149 | 1 | **0.042** |
| Lifetime alcohol misuse | -3.372 | 1.740 | 3.756 | 1 | 0.053 |
| Lifetime number of hospitalizations | -0.237 | 0.127 | 3.488 | 1 | 0.062 |
| Polarity at onset | 2.363 | 1.284 | 3.385 | 1 | 0.066 |
| DMR106540 | -0.300 | 0.172 | 3.057 | 1 | 0.080 |
| Lithium as first MS | -1.467 | 0.997 | 2.164 | 1 | 0.141 |
| Lifetime cannabis misuse | 1.424 | 1.170 | 1.480 | 1 | 0.224 |
| Constant | 37.748 | 16.686 | 5.118 | 1 | 0.024 |

Variables ordered from lowest to highest p values

DMR: Differentially Methylated Region, BD: Bipolar Disorder, SE: Standard Error, MS: Mood Stabilizer
